# *Acanthamoeba* Encephalitis in Patient with Systemic Lupus, India

**DOI:** 10.3201/eid1206.060087

**Published:** 2006-06

**Authors:** Charudatt G. Shirwadkar, Rohini Samant, Milind Sankhe, Ramesh Deshpande, Shigeo Yagi, Frederick L. Schuster, Rama Sriram, Govinda S. Visvesvara

**Affiliations:** * P.D. Hinduja National Hospital and Research Centre, Mumbai, India;; † California Department of Health Services, Richmond, California, USA;; ‡ Centers for Disease Control and Prevention, Atlanta, Georgia, USA

**Keywords:** *Acanthamoeba*, encephalitis, systemic lupus erythematosus, dispatch

## Abstract

We report a fatal case of encephalitis caused by *Acanthamoeba* in a 24-year-old woman from India with systemic lupus erythematosus. Diagnosis was made by identification of amebas in brain sections by immunofluorescence analysis and confirmed by demonstrating *Acanthamoeba* mitochondrial 16S rRNA gene DNA in brain tissue sections.

*Acanthamoeba* spp. are free-living amebae that cause granulomatous amebic encephalitis (GAE), most often in immunocompromised hosts, including HIV/AIDS and organ transplant patients and those receiving immunosuppressive medication (*1*). These organisms have also been associated with amebic keratitis, mainly in contact lens wearers, as well as with cutaneous, nasopharyngeal, and disseminated infections and amebic encephalitis.

Amebic encephalitis results from the hematogenous spread of the amebae from the initial portals of entry (skin, respiratory system) to the brain parenchyma. GAE is found worldwide (*1*); it develops as a subclinical infection and is difficult to diagnose because of vague symptoms. It is usually identified in a biopsy specimen of brain lesions or during postmortem examination.

Opportunistic infections are increasing because of HIV/AIDS, chemotherapeutic treatments, organ transplant procedures, and debilitating diseases. Although HIV/AIDS is under tenuous control in the industrialized world, it is a burgeoning public health crisis in the developing nations of Africa, Asia, and the Indian subcontinent. Amebic encephalitis cases likely are undetected in both industrialized and nonindustrialized nations. Acquainting clinicians with GAE is important so that they may include amebic encephalitis in differential diagnoses. We report a fatal case of *Acanthamoeba* encephalitis in a patient from India who was being treated for systemic lupus erythematosus with corticosteroids and methotrexate.

## The Case

The patient was a 24-year-old woman with a 2-year history of systemic lupus erythematosus, a history of central nervous system (CNS) involvement in the recent past, and autoimmune hemolytic anemia. She was taking chloroquine, prednisolone, and methotrexate. A month before her admission to the hospital, low-grade fever, joint pains, facial rash, and mouth ulcers developed. A day before admission she had generalized tonic clonic convulsions that progressed to status epilepticus, followed by loss of consciousness. On admission, she had a temperature of 40°C, pulse 110/min, blood pressure 126/80 mm Hg, and neck stiffness. She was stuporous and withdrawing to pain. Her cerebrospinal fluid (CSF) levels were the following: protein 174 mg/dL, glucose 42 mg/dL, erythrocytes 1/mm^3^, and leukocytes 1/mm^3^. Gram-stained CSF smears showed no bacteria, and CSF cultures were negative for bacteria and fungi. She was treated with ceftriaxone, phenytoin, and pulse steroids. On her second day in the hospital she was intubated for airway control. By that evening her pupils became small, quadriparesis developed, and she became deeply comatose.

**Figure 1:**
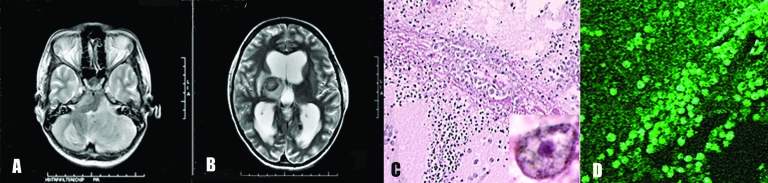
A) Magnetic resonance imaging (MRI) of the patient's brain showing a large lesion in the left cerebellar hemisphere. B) MRI in a different plane taken at the same time. Lesions are evident in the right thalamus and the right half of the pons. C) Blood vessel in brain parenchyma with large numbers of *Acanthamoeba* in the perivascular space (hematoxylin and eosin stained, magnification ×100). Inset, higher magnification (×1,000) showing nuclear morphology of the ameba. The dark-stained ameba nucleus with a central nucleolus is distinctive. D) Immunofluorescent staining of perivascular brain tissue showing many amebae (magnification ×100).

Magnetic resonance imaging (MRI) showed multiple hypointense necrotic lesions of varying sizes on T2-weighted imaging. These lesions involved the supra- and the infratentorial compartments, with the largest lesion in the left cerebellar hemisphere (Figure 1A). Ascending transtentorial herniation, significant mass effect, distortion of the brainstem and fourth ventricle, moderate supratentorial ventricular dilatation, and associated sulcal/meningeal enhancement occurred. Similar lesions of varying intensity and size were seen in bilateral cerebral hemispheres, left caudate head, right thalamus, and right half of the pons (Figure 1B). Three-dimensional multivoxel spectroscopy through the left basal ganglia and right thalamic lesion showed elevated lipid areas with increased lactate in the basal ganglia. Choline levels were normal or minimally increased.

Emergency posterior fossa craniectomy and foramen magnum decompression were performed. The left cerebellar lesion had poor demarcation from the adjacent normal cerebellar tissue and necrotic material in the cavity. The tissue was soft, discolored with gray-black spots, and hyperemic. An external ventricular drain was placed, and dexamethasone treatment was started. However, the patient's condition continued to deteriorate and she died after 5 days.

Histopathologic analysis of CNS lesions showed foci of hemorrhagic necrosis. There was a modest chronic inflammatory exudate composed mainly of lymphocytes, monocytes, a few plasma cells, and occasionally a few polymorphonuclear leukocytes. Amebic trophozoites were abundant, mainly around blood vessels, but few cysts were seen (Figure 1C). Amebae were identified by a nucleus containing a large central nucleolus (Figure 1C, inset). Formalin-fixed, paraffin-embedded sections were tested with rabbit antibodies to *Acanthamoeba* spp., *Balamuthia mandrillaris*, and *Naegleria fowleri*. Amebae were identified as *Acanthamoeba* on the basis of their reactivity in immunofluorescence analysis (Figure 1D).

DNA was extracted for amplification and sequencing from formalin-fixed brain tissue sections mounted on slides. Sections were deparaffinized, scraped from slides, suspended in lysis buffer, and tested by polymerase chain reaction (PCR) as previously described (*2*).

**Figure 2:**
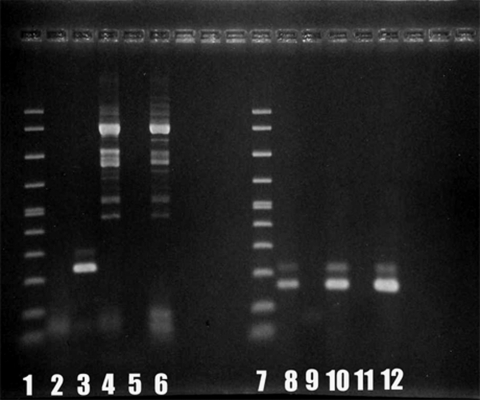
Results of polymerase chain reaction showing 230-bp and 161-bp bands for *Balamuthia* (lanes 1–6) and *Acanthamoeba* (lanes 7–12) mitochondrial 16S rRNA gene DNA. Lane 1, molecular mass marker; lane 2, absence of *Balamuthia* DNA in patient brain tissue (5-μL sample); lane 3, positive control (*Balamuthia* DNA); lane 4, negative control (*Acanthamoeba* DNA); lane 5, negative control (water); lane 6, absence of *Balamuthia* DNA in patient brain tissue (1-μL sample); lane 7, molecular mass marker; lane 8, *Acanthamoeba* DNA isolated from patient brain tissue (5-μL sample); lane 9, absence of *Balamuthia* DNA isolated from amebae in patient sample; lane 10, positive control (*Acanthamoeba* DNA); lane 11, negative control (water); lane 12, *Acanthamoeba* DNA isolated from patient brain tissue (1-μL sample).

The primer set Aca16Sf1010 (5´-TTATATTGACTTGTACAGGTGCT-3´) and Aca16Sr1180 (5´-CATAATGATTTGACTTCTTCTCCT-3´), which was designed to give an amplimer of 161 bp, was used for detecting *Acanthamoeba* DNA (*3*). Testing for *Balamuthia* DNA was also included because this ameba can cause GAE (*2*). PCR results were positive for *Acanthamoeba* DNA (Figure 2, lanes 7–12) but negative for *Balamuthia* DNA (Figure 2, lanes 1–6).

## Conclusions

Amebic encephalitis is caused by 3 different amebae: *Acanthamoeba* spp, *B*. *mandrillaris*, and *N*. *fowleri*. Since *Acanthamoeba* spp. and *B*. *mandrillaris* have similar forms and cause subacute, prolonged clinical courses, they have been misidentified in histopathologic examination of brain tissue (*4*). *N*. *fowleri* causes primary amebic meningoencephalitis, a fulminant disease often fatal within 7 to 10 days and usually associated with swimming or other water-related activities of otherwise immunocompetent persons, usually children or young adults (*1*). *Acanthamoeba* spp. are ubiquitous in soil and water and are found in the home environment, including water taps and sink drains, flowerpot soil, and aquariums. In healthcare settings, they have been isolated from hydrotherapy baths, dental irrigation equipment, humidifiers, cooling systems, ventilators, and intensive care units (*1**,**5*).

Encephalitis caused by *Acanthamoeba* is almost always fatal because of difficulty and delay in diagnosing the disease and lack of optimal antimicrobial therapy (*5*). The portal of entry of the ameba may be a break in the skin or the respiratory tract by inhalation of wind-blown cysts, with subsequent spread to the CNS through the circulatory system (*1*). In our patient, the portal of entry could not be determined.

Initial symptoms of GAE are vague and may mimic neurocysticercosis, tuberculoma, or brain tumor. Histopathologic analysis is the most common means of detecting amebae, but their appearance is often unfamiliar to pathologists, and they may be overlooked or misidentified (*1*). Either electron microscopy or immunostaining of brain tissue is necessary to distinguish *Acanthamoeba* spp. from *Balamuthia* (*1*). *Acanthamoeba* in our patient was identified by immunofluorescence testing using rabbit antibodies to *Acanthamoeba* (*1**,**5*). PCR was also used to detect *Acanthamoeba* DNA in brain tissue.

In almost all cases, persons with *Acanthamoeba* infections are immunocompromised (*1*). *Acanthamoeba* infections in persons with lupus erythematosus who were being treated with corticosteroids have been reported (*6**–**9*). Corticosteroids, besides reducing inflammation, impair the immune response, facilitating infection and disease caused by *Acanthamoeba* (*10*). In fixed brain tissue, *Acanthamoeba* and *B*. *mandrillaris* are similar and both form cysts in tissues (*4**,**5*). PCR has been used to identify ameba DNA in brain tissue and CSF of persons suspected of having balamuthiasis (*2*) and in brain tissue of a patient with primary amebic meningoencephalitis caused by *N*. *fowleri* (*11*).

In conclusion, *Acanthamoeba* infection was demonstrated by hematoxylin and eosin and immunostaining of brain tissue and PCR detection of ameba mitochondrial DNA in brain tissue. Treatment with corticosteroids and methotrexate likely facilitated development of amebic infection by compromising the patient's immune system.
